# “No sufro, estoy bien/I am not suffering, so I am doing OK”: A mixed method exploration of individual and network-level factors and Type 2 Diabetes Mellitus (T2DM) among Mexican American adults in New York City

**DOI:** 10.1371/journal.pone.0295499

**Published:** 2024-01-19

**Authors:** Karen R. Flórez, Neil S. Hwang, Maria Hernandez, Sandra Verdaguer-Johe, Kamiar Rahnama Rad

**Affiliations:** 1 Environmental, Occupational and Geospatial Sciences Department, City University of New York (CUNY), CUNY Graduate School of Public Health and Heath Policy, New York, NY, United States of America; 2 Center for Systems and Community Design, City University of New York (CUNY), CUNY Graduate School of Public Health and Heath Policy, New York, NY, United States of America; 3 Business and Information Systems Department, City University of New York, Bronx Community College, Bronx, NY, United States of America; 4 El Instituto: Institute of Latina/o, Caribbean and Latin America Studies of College of Liberal Arts and Science, University of Connecticut, Storrs, CT, United States of America; 5 Department of Community Health and Social Sciences, CUNY Graduate School of Public Health and Health Policy, New York, NY, United States of America; 6 Paul H. Chook Department of Information Systems and Statistics, City University of New York, Zicklin School of Business Baruch College, New York, NY, United States of America; University of Glasgow, UNITED KINGDOM

## Abstract

**Introduction:**

The prevalence of type 2 Diabetes Mellitus (T2DM) is 2–3 times greater among Mexican Americans than non-Latino whites, and Mexican Americans are more likely to develop T2DM at younger ages and experience higher rates of complications. Social networks might play a crucial role in both T2DM etiology and management through social support, access to resources, social engagement, and health behavioral norms.

**Objective:**

To quantitatively identify the social network features associated with glycated hemoglobin (HbA1c) in a community sample of Mexican immigrants residing in New York City, and to explore the extent to which these quantitative findings converge with qualitative narratives of their lived experiences.

**Methods:**

This study used a convergent mixed methods design. To collect personal network data, we used EgoWeb, and obtained 1,400 personal network ties from 81 participants. HbA1c readings were collected using dried blood spots and categorized according to the laboratory criteria of the American Diabetes Association. Additional survey data were collected using Qualtrics software. To investigate the significance of the network-level factors after accounting for the socioeconomic and demographic individual-level factors that the literature indicates to be associated with T2DM, we used a multiple regression model on quantitative data sources. For the qualitative portion of the study, we selected a subset of individuals who participated in the quantitative portion, which represented 500 personal network ties from 25 participants. We conducted in-depth interviews guided by the visualization of these ties to explore who was helpful or difficult in managing their health and health behaviors.

**Results:**

Individual-level indicators associated with lower HbA1c scores were body mass index (β = -0.07, p<0.05), and healthy eating index scores (β = -0.03, p<0.02). The network-level predictor associated with higher HbA1c levels was the percentage of diabetic alters in the network (β = 0.08, p <0.001, with a 25% increase in the percentages associated 2.0 change in HbA1c levels. The qualitative data highlighted that most of the diabetes-related information diffused through the social networks of our participants was related to dietary practices, such as reducing sugar and red meat consumption, eating out less, and reducing portion sizes. Notably, even among those with elevated levels and diabetes-related health complications, HbA1c was not considered a part of the lay descriptions of good health since they were not “suffering.” Participants regarded doctors as the ultimate authority in diabetes care, even if they had supportive members in their personal networks.

**Conclusion:**

Our study provides quantitative evidence for the significant role of diabetic network members in the etiology and management of T2DM among Mexican Americans. Our qualitative findings suggest important ley terms for T2DM management and the importance of physicians, which could be included in in future social networks studies seeking to diffuse diabetes-related health information for T2DM prevention and management efforts in this population.

## Introduction

The trends and prevalence of type 2 Diabetes Mellitus (T2DM) in Latinos residing in the United States are a major public health concern. The age- and sex-adjusted prevalence of diabetes among Latinos is 19.1% compared to 12.1% among non-Latino whites [1–3], and they are at a higher risk of inferior T2DM outcomes, such as amputations, end-stage kidney disease, and death [4]. Among Latino adults, the highest prevalence is found in Mexican Americans (21.7%) [1, 3] and they have a greater risk of complications associated with T2DM, particularly retinopathy and kidney disease [4].

Effective T2DM management requires controlling blood sugar levels, which can be measured by glycated hemoglobin A1C [Hba1c] and tests for the percentage of red blood cells with sugar-coated hemoglobin in the past three months [5]. However, Latinos have a lower prevalence of regular glucose monitoring than their white counterparts [6–10]. Poor T2DM management is associated with individual risk factors such as lack of health insurance. This situation is more common among Mexican Americans than among other Latinos and is linked to poorer health outcomes [11]. Healthcare expansions for low-income populations have been associated with an increased probability of diabetes diagnosis and uptake of medication regimens, however, long-term diabetes-related outcomes have been difficult to achieve [12]. This may be because of non-adherence related to issues beyond cost, such as faith in diabetes medications [13]. To be sure, cost plays a role, as research shows that income predicts adherence among Mexican Americans with T2DM, but there is also evidence that larger neighborhood-level factors may play a role in nonadherence due to beliefs about medications [11].

Another crucial aspect of T2DM management is maintaining a healthy diet, which can be difficult due to various factors such as material conditions (e.g., cost of food, access, and availability) and complex social and cultural factors (e.g., traditional dietary habits and obesogenic environments). Adapting to a healthy eating pattern requires individuals to overcome a range of social, economic, and cultural obstacles that often serve as a barrier to behavior change. Studies have shown the efficacy of nutrition education programs and interventions in increasing awareness and understanding of the necessary dietary changes for successful T2DM management, when culturally tailored approaches are employed [14]. However, attaining sustained individual-level dietary behavior change that leads to improved T2DM long-term outcomes has proven challenging, even with programs tailored to Mexican Americans [15]. Dietary changes such as limiting the use of traditional food items are difficult for any group; however, for Mexican Americans, a typical T2DM diet restricts many important food staples, including tortillas and rice [16]. This makes adopting the diet a difficult lifestyle adjustment not only for individuals with T2DM but also for their complex social system, which includes family members and acquaintances both inside and outside the home [14].

Despite the complexity and multi-level nature of factors that could help engage individuals with T2DM in self-care behaviors [14], research has largely focused on individual-level factors based on theories of individual change [14, 17]. Specifically, among Mexican Americans with T2DM, one area of research has relied heavily on the theory of locus of control, which posits that “external” control orientations may lead to poorer T2DM outcomes, as patients may believe that the disease is beyond their control [18]. Intervention studies using this theory postulate that this psychological orientation is associated with patients taking less action, resulting in poor outcomes [19–22]. However, there is a lack of evidence that this is the case [23]. Research on Mexican Americans with T2DM has also focused on acculturation, which refers to the process by which immigrants adopt the norms, values, beliefs, and behaviors of the dominant group [24–26]. Despite a consistent relationship showing that lower levels of acculturation are associated with lower T2DM prevalence among non-Mexicans, including other Latinos [15, 27–29], those in transition or the “middle” phase of the acculturation process complicate this relationship in samples of Mexican immigrants [30, 31]. Several factors drive these potential differences, including levels of human and social capital, reasons for migration and subsequent opportunities for successful integration, and transnational social network ties to Mexico [32].

However, despite the evidence that underscores the importance of sociocultural factors in the study of T2DM, far fewer studies have taken a social-network perspective [17]. Interventions among Latinos with T2DM, approached from a social network perspective, suggest that leveraging the knowledge and experience of network members improves T2DM in the short term (<6 months) [33–38]. However, current studies have mainly focused on either dyads or peers, and there has been limited research on how social networks can be leveraged for long-term outcomes. To fully harness the potential of social networks for T2DM management, it is crucial to understand the structural features and attributes of personal networks among Mexican Americans. By gaining a deeper understanding of how social networks operate within this population, interventions can be tailored to optimize diabetes management and improve long-term outcomes. A large community of researchers across disciplines has developed useful theoretical frameworks to understand how ties can be viewed as conduits through which resources (e.g., information and support) can flow to and from. Public health research from a network perspective has largely focused on infectious diseases and tobacco use [39], with a focus on how knowledge and behavior can be powerfully spread across interpersonal ties [40, 41] as well as how networks comprising such ties can amplify the spread [41].

The effect of a person’s behavior on their social network has significant implications for public health and has been explored through various theoretical perspectives in multiple disciplines [39, 42]. One widely recognized theory in public health is the theory of diffusion, which postulates that individuals can influence each other through their interactions and develop similar beliefs or become aware of similar information [42, 43]. Social capital studies represent another rich tradition, in which the flow of resources or network benefits depends on varying degrees of access and permission. Within each of these traditions, most studies take a whole network approach and focus on the pure effects of the network structure (e.g., equivalence, centrality) on an outcome [42, 43]. Another set of studies explained networks from an egocentric perspective. Sometimes also referred to as personal-network studies, in such research, the focal point of the researcher is to understand the “ego” and her set of ties to others in their network called the “alters” [43]. These studies have also utilized diffusion and social capital frameworks, but it is important to note that they typically explore issues beyond structure, such as attributes of the ego, as well as attributes of the alters [42].

Focusing on the attributes of alters is an important step in characterizing the personal networks of Mexican Americans to elucidate several issues within the theoretical frameworks of social capital and diffusion. First, there is an unexplored aspect of social homogeneity as it relates to the process of acculturation for Mexican Americans. That is, the acculturation process is associated with T2DM and important self-care behaviors, but scholars have conceptualized this as an individual-level process. Almost no work has examined the acculturation attributes of a personal network in which an immigrant person is embedded. Second, there is a lack of nuanced understanding of the diffusion process by locus of agency. That is, it is unclear whether Mexican American egos adopt from their alters in a largely active or passive way and whether the alters employ active processes (e.g., through social control) or mimetic processes (e.g., through habitual practices). Third, contextual details of the lived experiences of Mexican American immigrants could shed light on how migration can disrupt social networks and relationships, which can be highly destructive to social capital, especially among those in transition or in the “middle” of the acculturation process. This mixed-method study aims to shed light on these complex issues within the context of T2DM and self-care behaviors among Mexican Americans in New York City, using qualitative and egocentric network data. Despite the importance of social networks in shaping health behaviors, few studies have explored their role in promoting long-term behavior change among this population. To address this gap, this mixed-method study asked the following research questions (1) What social network features are associated with HbA1c levels among a community sample of Mexican immigrants in New York City? (2) How do these quantitative features converge with qualitative narratives of their lived experiences? Given that over 61% of all Latinos are of Mexican origin, with diverse cultural backgrounds and deep roots in Latin America [44], understanding the social determinants of health within this population has significant public health implications.

## Methods

We used a mixed-methods approach, employing a convergent design [45], with the quantitative phase preceding the qualitative phase. The study involved the same cohort of participants in both the stages, ensuring consistency and coherence across data collection and analysis, as described below.

*Quantitative sample*: 1,620 personal network ties were derived from 81 self-identified Mexican American adults recruited from a large Catholic Church in the Bronx, New York. Specifically, the church liaison identified potential prayer and activity groups with large numbers of Mexican American congregants, and we also used telephone rosters to identify potential participants. Three trained bilingual research assistants contacted participants in person or via telephone and provided them with an explanation of the details of the study. After screening for eligibility, interested individuals consented to participate using a protocol approved by the City University of New York Human Subjects Institutional Review Board (IRB# #2018–1081). The same research assistants scheduled all data collection at the participants’ preferred time and location for convenience. None of the research assistants had previous relationships with any of the participants. However, before the interview process began and to put the participants at ease, they occasionally shared details about themselves such as their educational background (one held an MPH and was pursuing additional training in a doctoral program in public health; the other two held BS degrees and one was obtaining additional training in a Master’s program in public health nutrition), as well as their nationality (e.g., Mexico; Spain; Poland). Data were collected in person between January 2019 and June 2019 in either English or Spanish, depending on the participant’s preference.

*Quantitative data*: Three different approaches were used to collect the various forms of quantitative data used for this analysis. First, EgoWeb was used to collect interviewer-assisted personal network interviews that consisted of three sections: (i) generating names of people in the respondents’ personal social networks (alters), (ii) collecting information about each alter (network composition), and (iii) eliciting information about the relationship between each unique pair of network alters (network structure) [46]. We asked respondents to name (by first name only) 20 individuals whom they knew, who knew them, and with whom they had contact sometime in the past 6 months, using standard free listing techniques employed in personal network data collection among vulnerable populations [47]. Eliciting 20 alters has been found to be enough data to capture variation of the network structure while still maintaining respondent burden at manageable levels [48]. Contact included face-to-face, phone/text, mail, e-mail, social media (e.g., Facebook), and research assistants probed participants to think about additional social groups (e.g., church members) to reach the 20 alters, which all participants were able to do. Secondly, Qualtrics [49] was used to collect interviewer-assisted survey data on the demographics and other individual-level characteristics of the participants, using standard measures when possible. Finally, whole dried blood spots from a finger prick were used to collect HbA1c levels, following the 2018 ADA Standards of Medical Care in Diabetes [50]. This minimally invasive approach for blood sampling can be performed in non-clinical settings by non-medically trained personnel [51]. Research assistants were trained to use universal health precautions and a common protocol to collect the blood spots from participants (six steps) and send the specimens to the lab (three steps) [52], ensuring data quality and participant safety. The software R (version 4.2.2) was used for statistical analysis [53].

*Power considerations*. Our primary aim was to determine whether it is feasible to conduct such a study with this population and to obtain a better understanding of the variability in the outcomes of interest and social network characteristics to design a larger-scale study. Our sample size of 70 was sufficient to detect effect sizes of 0.25 standard deviations, or larger, with a significance level of α = 0.05, and power of 0.8 (β = 0.8).

### Dependent variable

#### Hemoglobin A1c (HbA1c) %

A dried blood spot test for HbA1c was analyzed by an independent laboratory, and results were sent back as HbA1c % for each participant. Higher HbA1c levels are associated with an increased risk of diabetes complications [5]. Following standard ranges [5], we defined normal as below HbA1c, prediabetes as HbA1C levels between 5.7% and 6.4%, and diabetes as HbA1c levels above 6.4%. These definitions were then used to classify the participants’ HbA1c levels and investigate their association with other variables of interest.

### Individual-level measures

#### Household food security

Measured using the six-item U.S. Department of Agriculture Food Security (USDA) Survey. Responses were dichotomized according to according to USDA guidelines [54], with participants who reported very low, low, or marginal food security categorized as having low food security, while those who did not report such levels of food security were categorized as having high food security.

#### Health insurance coverage

We asked participants whether they had any health insurance coverage that helped them pay for their medical bills, including Medicaid. These responses were also dichotomized and in line with other studies with Latino immigrants [55, 56], with participants who reported having Medicaid, Medicare, or private insurance categorized as having health coverage (yes), while those who reported not having any insurance were coded as not having health insurance (no).

#### Educational attainment

Participants were asked to rate their highest level of education on a 7-point scale, which ranged from (1) 6^th^ grade or less (elementary school) to (7) some graduate or graduate degree. For those participants who attended school outside the U.S., we asked them to identify the U.S. equivalent. We then dichotomized the responses, classifying participants who reported 11^th^ grade education or below as “less educated”, and those with high school education or higher (including the General Educational Development test, or GED) categorized as “more educated”.

### Remittance

We asked the participants whether they had sent money to their families and relatives in Mexico. Response options were ‘yes,’ “‘ no,’” ‘ refused,’ and ‘do not know.

### Network-level measures

#### Percentage of alters eating American food

To assess the extent to which the ego’s network members consumed American food, we asked the ego the question ‘How much do you agree or disagree that Person X eats American food most of the time?’ The possible responses were ‘Strongly Agree’, ‘Agree’, ‘Disagree’, and ‘Strongly Disagree’. Responses indicating agreement (‘Strongly agree’ and ‘agree’) were coded as network members who mostly eat American food. We then calculated the proportion of network members (out of 20 total) who fell into this category. This measure was included to help participants distinguish between alters who consume a traditional Mexican diet and those who consume a Standard American Diet, which is characterized by foods high in calories and low in nutrients, such as sugar-sweetened beverages and processed foods [57].

#### Percentage of alters who encourage the ego’s health

Assessed via two questions (1) “In the past 6 months, who helped you or encouraged you to have a healthy lifestyle, to eat healthy foods or to be active?” and (2) “In the past six months, who did you go to for information, advice, or suggestions about your health, your family’s health, or other health concerns?” Network members that provided any type of health-related support were coded as “support providers”. The percentage of support providers was calculated by dividing the number of support providers by the total number of network members (N = 20).

#### Percentage of alters with diabetes

The participant was asked “Of all of the people on your list, who do you know that has been diagnosed with diabetes?” The number of alters identified was divided by the total number of network members (N = 20) to calculate the percentage of alters with diabetes.

#### Percentage of alters who eat American food and eat with ego

Among the participants who eat American food, we identified the number of alters who also responded with the ego’s name to the following question: “In the past 6 months, who did you share meals or snacks with on a regular basis (at least a few times a month or more)? This could include eating at your home or their home, or eating out.” This number was then divided by the total number of network members (N = 20) to calculate the percentage of alters who eat American food and eat with the ego.

#### Percentage of alters who are diabetic and eat with ego

Among the participants who are diabetic, we identified the number of alters who also responded with the ego’s name to the following question: “In the past 6 months, who did you share meals or snacks with on a regular basis (at least a few times a month or more)? This could include eating at your home or their home, or eating out.” This number was then divided by the total number of network members (N = 20) to calculate the percentage of alters who are diabetic and eat with the ego.

### Confounders

Age, Standard Acculturation Stress Scale [58], where scores (between 1 and 9, with higher scores indicating stronger acculturative stress), years of residency in the United States (positive integer), and family income (positive integer). We also included general health (good vs. poor), marital status (between 1 and 6 with 1 = single, never married, 2 = married, 3 = living with partner but not married, 4 = divorced, 5 = separated, 6 = widowed), diabetes under control (yes vs. no), US citizenship (yes vs. no), sex (female vs. male), alcohol consumption (yes vs. no), smoking (yes vs. no), working (yes vs. no), objectively measured Body Mass Index (BMI), Healthy Eating Index (HEI) derived from 24-hour dietary recalls (scores 0 to 100, with higher scores representing diets that are more strongly aligned with the USDA dietary recommendations) [59], “IPAQ” (International Physical Activity Questionnaire) that measures leisure-time physical activity with moderate intensity (e.g., leisure cycling), and vigorous intensity (e.g., running or aerobics) (1:low, 2:moderate, 3:high).

### Quantitative analysis

For confounder variables, we identified socioeconomic and health-related individual-level factors that the literature indicates are associated with T2DM [30, 31, 60–70]. We then fitted multiple linear regression models to examine the significance of individual- and network-level factors in explaining the variability in HbA1c levels and provide a summary of regression model diagnostics as S1 Table. We excluded one observation because it was a single outlier born in the U.S. We then removed 10 observations that had missing data for one or more of the resulting 24 covariates and estimated a multiple regression model using the remaining 70 observations. However, we assessed missingness patterns by examining the distributions of variables with missing values against those that are fully observed using pairwise margin plots and concluded that the missingness pattern in the dataset was consistent with the MCAR (missing completely at random) assumption to justify listwise removal of missing observations.

### Qualitative sample, data collection, and analysis

Using the quantitative social network data to derive our qualitative sampling frame, we categorized potential participants by “low” “moderate” and “high” connectivity. Our approach was intended to identify information-rich cases, which is a purposive sampling method employed by descriptive studies seeking broad insights into a phenomenon [71]. The sample of 25 was deemed to have sufficient information power, given our narrow aim and complex conversations with participants regarding the role of social networks [72]. No participant that chose to partake in the qualitative portion of the study dropped out, but those that were approached to participate refused mostly because they lacked time. Data was collected face-to-face by the one of the female bilingual research assistants who performed the quantitative data collection and who has lived experienced as an immigrant Latina in NYC, so they built on the trust established during their previous interaction during the quantitative interview. For those they interacted with for the first time, they approached them with care and compassion given their deep understanding of dynamics when working with community of color.

All interviews were performed using the same semi-structured interview guide, which leveraged the visual representation of the participant’s personal network. The semi-structured interview guide was designed in the spirit of hermeneutic or interpretative of phenomenology given the framework’s usefulness in exploring first-person experiences while still allowing for the researcher to have an active role in data collection [73]. Specifically, the bulk of the interview would center around the ego’s experience of alters who they identified as people they go to for health advice, as well as those that help their efforts at healthy eating and physical activity. There was also a section specifically on the alters whom the ego shares meals or snacks with, and the questions for each alter explored (1) the extent to which the way they eat with alter X differs from the way they usually eat (2) what type of food or snacks do they have with alter X (3) Are there other people around when you eat with alter X (4) How different are these meals and snacks that you have here in the United States from what you would have if you were living in Mexico? Because not all members were supportive, there was also a section of the interview when participants could also reflect on their lived experience regarding lack or difficulty in obtaining support from their network around these topics. All these specific questions were design to situate the participant within the visual depiction of their network, but less scripted probes were used to elicit narratives around certain alters and elicit participants’ meaning around how their personal network affected their health. Memos were written after each interview to capture reflections, which are also in line with the interpretive phenological approach [73]. These as well as each interview was recorded verbatim and reviewed by the main interviewer for accuracy, though the transcript was not returned to participants for comments. Interviews were conducted in Spanish except for one conducted in English, and all interviews took about an hour and half to complete. Most interviews took place in an area of the church’s community center with some privacy (e.g., classroom) and most had no one with them during the interview except for a few that brought small children or elderly parents with them.

Using the qualitative and mixed-method data software program Dedoose (version 9.0.17) [74]. the first cycle of coding was performed by the two research assistants who collected either the quantitative or qualitative data given their close proximity to the participants. They were joined by an additional bilingual research assistant obtaining their doctoral degree in cultural anthropology but who was not involved in any of the data collection. A series of iterative steps were taken as 3-person team to develop the code book [75]. Specifically, the steps taken included (1) development of an initial code list derived from etic concepts, emic themes, or both, (2) two or more coders independently coded the same text based on a common definition, (3) results of the coding were compared for consistency of text segmentation and code applications, (4) the codebook was reviewed to determine whether the inconsistencies were due to coder error, and (5) once problems were clarified, all previously coded text was reviewed and if necessary, recoded so that it was consistent with revised definition. The inter-coder agreement process entailed a detailed segment-by-segment review that includes assessments of consistency in defining the beginning and end of segments as well as the application of codes within segments. Cohen’s Kappa was calculated for a few codes, with some yielding higher scores between coders than others (e.g., for codes developed for alters who helped the participant eat healthy was 0.60 for coder one and 0.53 for coder two; while for codes developed for alters who made it difficult to eat healthy it was 0.62 for coder one and 0.72 for coder 2). For that reason, any disagreements between coders were arbitrated by a third member and we refrained from further quantifying the degree of consensus as others have noted the drawbacks from this practice [76]. This was performed until no other codes emerged (i.e., the team reached thematic saturation) [77].

The second cycle of coding used cumulative coding methods and was performed by the first author, PI of the project with extensive experience in Latino health research including networks, and who also has lived experience as an immigrant Latina in NYC. Specifically, they evaluated numerous pages of coded text for each participant to generate a broad understanding of the data, and this was driven by the main qualitative research question of how do the quantitative features identified by the quantitative analysis converge with qualitative narratives of their lived experiences? This involved deep engagement with the qualitative data via reading, reflective writing, re-reading and re-writing of findings though all members of the interview, coding, and analysis team reviewed the results in an attempt to create a “hermeneutic circle” guiding our qualitative approach [73].

## Results

### Quantitative results

Table 1 shows the descriptive statistics for the sample according to HbA1c categories. Those with normal HbA1c levels were more likely to have high food security and health insurance and were more educated than those with abnormal HbA1c levels.

**Table 1:** Summary statistics for individual-level and network-level variables.

*Individual-level Variables*	Normal (*n =* 28)^a^	Prediabetes (*n* = 27)^b^	Diabetes (*n* = 15)^c^
Household Food Security			
High	21 (72%)	19 (70%)	6 (40%)
Low	7 (28%)	8 (30%)	9 (60%)
Health Insurance Coverage			
No	10 (35%)	13 (48%)	6 (40%)
Yes	19 (65%)	14 (52%)	9 (60%)
Education			
Less than 11^th^ Grade	13 (45%)	16 (59%)	10 (67%)
Completed High School	7 (24%)	6 (22%)	4 (27%)
Some College	9 (31%)	5 (19%)	1 (6%)
Sex			
Male	8 (29%)	6 (22%)	6 (40%)
Female	20 (71%)	21 (78%)	9 (60%)
General Health			
Poor or Fair	12 (43%)	15 (56%)	9 (60%)
Good, Very good, Excellent	16 (57%)	12 (44%)	6 (40%)
Working			
No	11 (39%)	11 (41%)	5 (33%)
Yes	17 (61%)	16 (59%)	10 (67%)
US Citizenship			
No	26 (93%)	26 (96%)	13 (87%)
Yes	2 (7%)	1 (4%)	2 (13%)
Drink alcohol			
No	11 (39%)	10 (37%)	6 (40%)
Yes	17 (61%)	17 (63%)	9 (60%)
Diagnosed with Diabetes or Sugar Diabetes			
No	28 (100%)	25 (93%)	13 (87%)
Yes	0 (0%)	2 (7%)	2 (13%)
Smoking			
Not at all	0 (0%)	0 (0%)	0 (0%)
Some days	27 (96%)	26 (96%)	15 (100%)
Everyday	1 (4%)	1 (4%)	0 (0%)
Remittance			
No	8 (29%)	9 (33%)	4 (27%)
Yes	18 (64%)	18 (67%)	11 (73%)
Refused to answer	2 (7%)	0 (0%)	0 (0%)
Marital Status			
Single, never married	9 (32%)	7 (26%)	3 (20%)
Married	8 (29%)	10 (37%)	8 (52%)
Living with partner, but not married	9 (32%)	3 (11%)	1 (7%)
Divorced	1 (4%)	2 (7%)	1 (7%)
Separated	1 (4%)	5 (19%)	1 (7%)
Widowed	0 (0%)	0 (0%)	1 (7%)
Age			
18–30	4 (14%)	5 (18%)	0 (0%)
31–40	13 (46%)	4 (15%)	0 (0%)
41–50	8 (29%)	11 (41%)	6 (40%)
51–60	3 (11%)	7 (26%)	6 (40%)
61–100	0 (0%)	0 (0%)	3 (20%)
Family Income			
Less than $9,999	9 (32%)	4 (14%)	5 (33%)
10,000–19,000	7 (25%)	8 (30%)	6 (40%)
20,000–29,000	5 (18%)	8 (30%)	1 (7%)
30,000–39,000	2 (7%)	2 (7%)	1 (7%)
40,000–49,000	2 (7%)	0 (0%)	0 (0%)
50,000–59,000	0 (0%)	1 (4%)	0 (0%)
60,000–69,000	1 (4%)	1 (4%)	2 (13%)
70,000–99,000	1 (4%)	2 (7%)	0 (0%)
More than 100,000	1 (4%)	1 (4%)	0 (0%)
Acculturation Score			
Median (IQR^1^)	4.0 (3.0, 5.0)	4.0 (2.0, 7.0)	4.0 (3.0, 5.0)
Mean ± SD^2^	4.4 ± 2.2	4.5 ± 2.4	4.2 ± 2.0
HEI Total Score			
Median (IQR^1^)	59.7 (51.2, 67.8)	53.9 (46.9, 60.4)	60.7 (51.9, 64.5)
Mean ±SD^2^	60.9 ± 10.1	53.5 ± 11.1	57.9 ± 14.7
IPAQ			
Median (IQR^1^)	2.0 (1.0, 3.0)	2.0 (1.0, 2.0)	1.0 (1.0, 2.0)
Mean ±SD^2^	2.0 ± 0.9	1.7 ± 0.8	1.6 ± 0.7
Years of residency in the U.S.			
Median (IQR^1^)	15.5 (9.8, 22.0)	24.0 (14.5, 28.5)	31.0 (19.5, 32.5)
Mean ± SD^2^	15.4 ± 9.1	20.9 ± 10.1	25.9 ± 11.4
BMI			
Median (IQR^1^)	28.3 (26.4, 31.0)	30.1 (25.7, 34.7)	32.5 (29.2, 33.7)
Mean ± SD^2^	29.1 ± 4.0	30.4 ± 6.3	31.1 ± 4.1
*Network-level Variables (Percentage in Network)*			
Alters eating American food			
Median (IQR^1^)	40 (15, 60)	50 (32, 70)	55 (35, 72)
Mean ± SD^2^	40 ± 31	51 ± 27	54 ± 27
Alters who encourage the ego’s health			
Median (IQR^1^)	20 (10,30)	10 (5, 15)	10 (5, 15)
Mean ±SD^2^	26 ± 24	16 ± 21	13 ± 14
Alters with diabetes			
Median (IQR^1^)	0 (0, 10)	5(0, 10)	10 (10, 17)
Mean ±SD^2^	5 ± 7	6 ± 6	14 ± 7
Alters with diabetes who eat with ego			
Median (IQR^1^)	0 (0, 0)	0 (0, 5)	0 (0, 5)
Mean ±SD^2^	2 ± 4	2 ± 3	3 ± 4
Alters who eat American food and eat with ego			
Median (IQR^1^)	5 (0, 20)	10 (5, 25)	10 (5, 20)
Mean ±SD^2^	13 ± 14	19 ± 20	14 ± 12

^a^ Normal: HbA1c < 5.7

^b^ Prediabetes: 5.7≤HbA1c ≤6.4

^c^ Diabetes: Hba1c >6.4

^1^ Interquartile range

^2^ Standard Deviation

Table 2 present the estimated coefficients and significance levels for each indicator in the multiple regression model. At the significance level of 0.05, the individual-level indicators associated with HbA1c levels were body mass index (BMI; β = -0.07) and healthy eating index (HEI) total score (β = -0.03).

**Table 2:** Coefficients for individual and network-level variables with HbA1c.

	Coefficient		(SE^1^)
*Intercept*	12.97	***	(2.61)
Household Food Security (High vs. Low)	-0.65	†	(0.35)
Health Insurance (Yes vs. No)	-0.56	†	(0.29)
Education (≤11^th^ grade vs High school graduate)	-0.31		(0.34)
Education (≤11^th^ grade vs. Some college)	-0.65	†	(0.38)
General Health (Yes vs. No)	-0.28		(0.27)
Acculturation Score (1 to 9)	-0.13	†	(0.08)
Has been diagnosed with diabetes (Yes vs. No)	-1.03	†	(0.57)
IPAQ (1, 2, or 3)	-0.05		(0.16)
HEI Total Score (0 to 100)	-0.03	*	(0.01)
Years of Residency in the U.S.	0.01		(0.02)
Working (Yes vs. No)	0.16		(0.37)
US Citizen (Yes vs. No)	0.12		(0.62)
Remittance (vs. Yes)			
No	0.34		(0.33)
Refused	0.53		(0.81)
Age	0.14		(0.19)
Marital Status (vs. Single, never married)			
Married	-0.19		(0.45)
Living with partner but not married	-0.40		(0.41)
Divorced	-0.84		(0.66)
Separated	-0.88		(0.57)
Widowed	0.36		(1.39)
BMI	-0.07	*	(0.03)
Sex (Male vs. Female)	0.13		(0.36)
Alcohol (No vs. Yes)	-0.49	†	(0.29)
Smoking (Someday vs. Everyday)	0.34		(0.89)
Family Income	-0.10		(0.07)
Percentage of alters eating American food	0.01		(0.01)
Percentage of alters who encourage ego’s health	-0.01	†	(0.008)
Percentage of alters with diabetes	0.09	***	(0.02)
Percentage of alters who eating American food and eat with ego	1.20		(1.16)
Percentage of alters with diabetes who eat with ego	-4.60		(4.44)
R^2^			0.89
Adjusted R^2^			0.47

**Fig 1:**
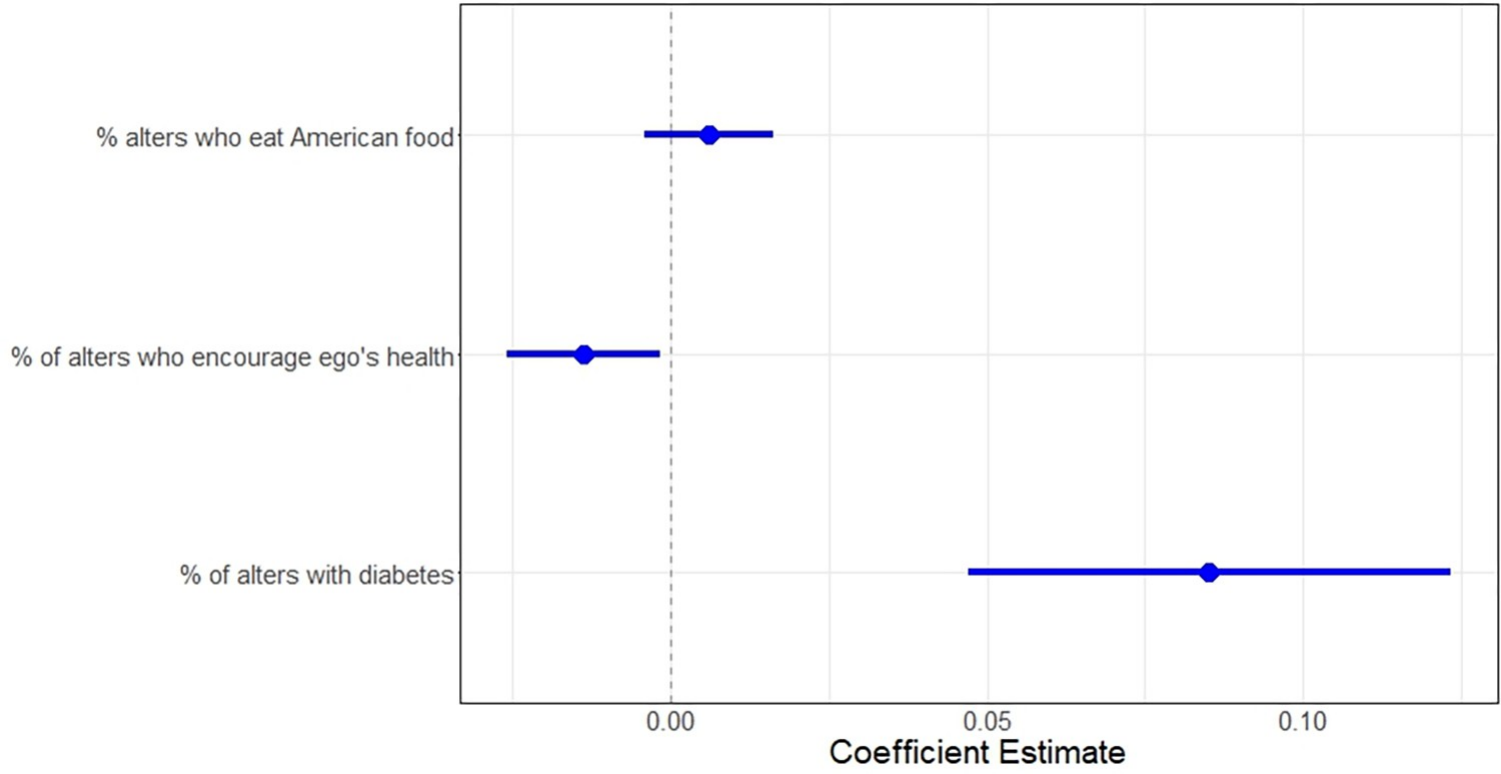
Close-up coefficient plot of network-level covariates.

Table 2 and Fig 1 show the estimated coefficients and significance levels for each network-level indicator in the multiple regression model. For a 25% increase in each network-level indicator (i.e., 5 more people with this attribute), we found at the 0.05 significance level that the percentage of alters who were diagnosed with diabetes was associated with an increase of 2.0.

### Integration with qualitative results

Given the convergent mixed-method design of our study, we analyzed the qualitative data in light of the relationships established in the quantitative analysis. As such, we were particularly interested in narratives discussing alters who were diabetic in the wider network and not just those in the “consumption” network (e.g., alters with whom the person eats with). We describe the level of social network connectivity and key demographic characteristics of the qualitative subsample by normal, prediabetic, and diabetic status in Table 3. Most of the subsamples had high levels of connectivity, and those with diabetes were older and had a higher proportion of health insurance and males relative to those categorized as normal or prediabetic. We included participants with normal and prediabetes hba1c levels because they still had alters in their personal social networks that were diabetic.

**Table 3:** Descriptive characteristics for qualitative sample.

	Normal *(n* = 8)	Prediabetes (*n* = 8)	Diabetes (*n* = 4) ^1^
**Level of Connectivity**			
High	5 (62.5%)	4 (50.0%)	2 (50.0%)
Moderate	3 (37.5%)	1 (12.5%)	0 (0%)
Low	0 (0%)	3 (37.5%)	2 (50.0%)
**Demographic Characteristics**			
Age	33.1 (3.1)	38.4 (4.8)	57.5 (2.7)
Female	5 (62.5%)	7 (87.5%)	1 (25.0%)
Household yearly income ≤$30K	8 (100%)	7 (87.5%)	3 (75.0%)
No HS diploma or GED	4 (50.0%)	4 (50.0%)	2 (50.0%)
Has health insurance	5 (62.5%)	4 (50.0%)	4 (100.0%)

^1^ n = 5 missing HbA1c data

**Fig 2:**
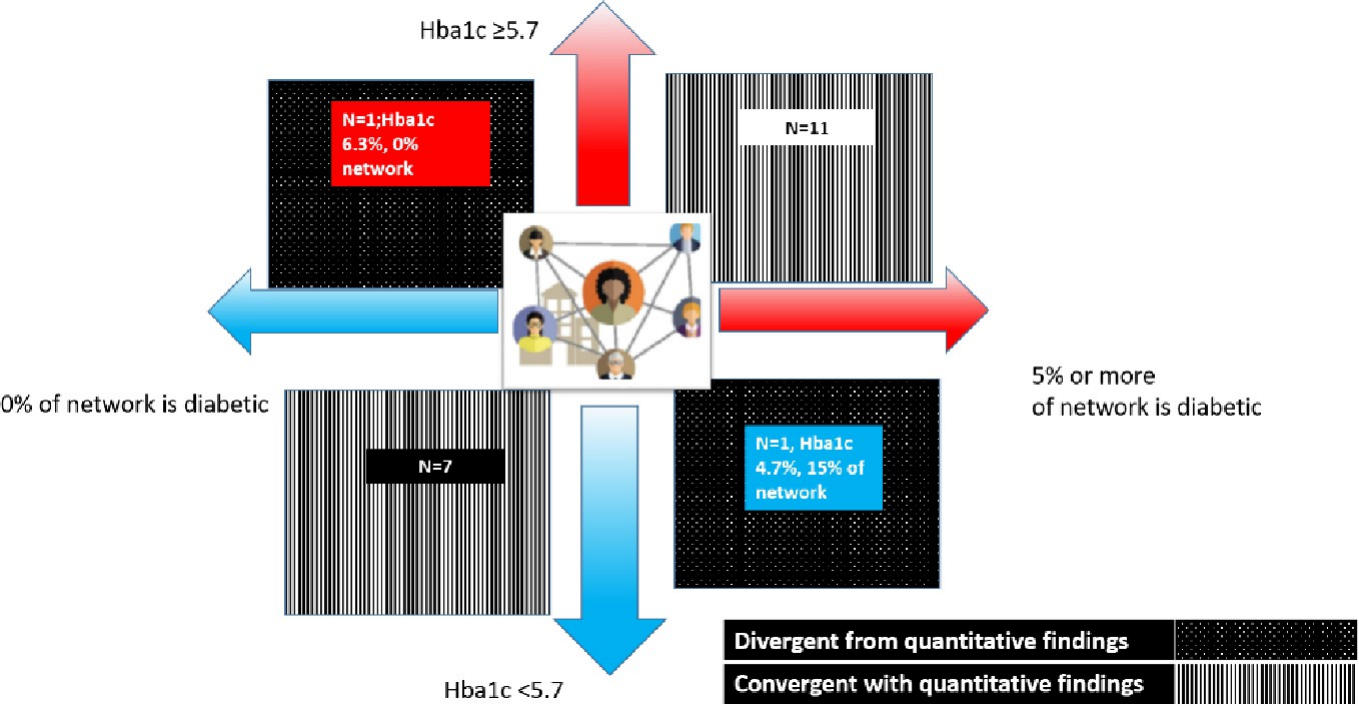
HbA1c levels on the y-axis and percentage of alters in the network that are diabetic on the x-axis.

To further differentiate the qualitative narratives of participants based on the quantitative findings of percentage diabetic, we illustrate the proportion of diabetic alters along with their own Hba1c score (if available) for the qualitative subsample of 25 participants (see Fig 2: HbA1c levels on the y-axis and percentage of alters in the network that are diabetic on the x-axis). This shows N = 18 participants who were convergent with the quantitative findings and (i.e., those with normal HbA1c levels and a low percentage of people in their network with diabetes, as well as those with high HbA1c levels and a high percentage of people in their network with diabetes) from those who were divergent (N = 2) (i.e., those with normal HbA1c levels and a high percentage of people in their network with diabetes, as well as those with high HbA1c levels and a low percentage of people in their network with diabetes). The analyses of the narratives from this convergent group highlighted the following themes.

## Convergent group: Elevated HbA1c levels and a higher percentage of alters with diabetes

### I am not suffering, so I am doing OK

Despite their elevated hba1c levels, these participants emphasized their ability to manage the condition well due to a lack of acute physical complications. Specifically, some concentrated on their ability to still work, get up early in the morning, and drive at night, as exemplified by the quotes below. Being able to engage in these physical actions, as well as their positive outlook while doing so, typified the reasons why their uncontrolled diabetes was not seen as a health issue that warranted medical attention.

**Table:** 

Participant 125, Hab1c 7%, 20% of network diabetic	I have the mentality of a person 20 or 25. I get up at 5am in the morning, I go up and down the stairs, and make tamales. My day starts at 5am and ends at 10pm and I feel fine. That is, I have energy and I do all sorts of things and I am keeping busy and trying to learn new things. “No sufro, estoy bien”: I am not suffering; I am doing well.
Participant 77, HbA1c 9.8%, 25% of network diabetic	[For example], they say that those with diabetes cannot see well [especially] at night. My sister’s husband, who is much younger than me, is [diabetic] and he no longer [feels comfortable] driving at night. I drive at night, at any time of the night if it is necessary. He cuts himself and the wound becomes red, and it takes a while to heal. Me, I cut myself and in three days it heals. [Therefore] I do not give myself [an insulin] shot…but I do not feel badly.

### Influence of personal network on diet

Participants narratives highlighted how network members shaped their diet, and not just those that they ate with on a regular basis. Most of the shaping came in the form of advice of what foods to avoid, which required changes such as reducing sugar and red meat intake, as well as portions and eating out. Sometimes the advice came from alters who struggled with their own elevated hba1c levels, though most participants referred to this as “high sugar.” For the most part, the diabetic alters highlighted in the exemplary quotes were not described as successful in their own efforts yet provided participants with a foreboding advice in the need to change their dietary habits. Of the few positive examples of successful management, was a participant 127 who described a diabetic alter that kept her weight under control by only eating homemade food (e.g., making her own salsa and tortillas) which in turn would save her money. But for the most part, participants described diabetic alters seeking help only after a major health scare, which reinforces this earlier theme of “I am not suffering so I am doing OK” as potentially normative behavior.

**Table:** 

Participant 17, HbA1c % 6.3, 15% of network diabetic)	Yes, we [my sisters and I] had very high sugar so we were [diagnosed] as diabetics like my mom…we were influenced by this idea of “[if you] eat [whatever you like] that nothing will happen; this is only one day in your life.” But I remember one day her sugar and her [blood] pressure dropped and we all agreed that we cannot accept this, and we have to be strong [against this advice]. So now we are changing little by little and it was really this scare that my mom had at the time. Right around that time our neighbor also suffered a scare because they had changed his [diabetes] medication because it was not working well. After this [he] started feeling short of breath, his stomach hurt, and he had some sort of allergy all over his body. He could no longer control this and said “No, no I also have to be [more] conscientious [of my health].” And because he works at a barbershop, he also had two or three clients that at the time had stopped seeing him, but it [turns out] they were dead because they had a heart attack because their sugar was very high. That really made an impression on him too, and he got scared and we started eating less in restaurants for example…
Participant 127, HbA1c 5.4%, 0% of network diabetic	Now that his sisters have been diagnosed with diabetes, now [my husband] has changed his tune. Now he says "no, let’s not eat that much sugar. It is better if we drink plain water."
Participant 134, HbA1c missing, 10% of network diabetic	Yes, [my son] has changed his eating habits because they told him he was pre-diabetic. I think because he works in security, it is also hard. But now he tells me "Mom, if I eat a pizza, I will at least get the one with vegetables."
Participant 67, HbA1c 5.9%, 20% of network diabetic	Well, my uncle, may God rest his soul, he was diabetic. He would always tell me to take care of myself. To not eat red meat, things with a lot of sugar. Definitely to stay away from sugar and avoid salt. He also told me about the importance of sleep to avoid getting sick. Therefore, he also helped me in that regard by giving me [health advice].
Participant 127, HbA1c 5.4%, 0% of network diabetic	[My sister] helped when they told me I had prediabetes. She told me “You have to take care of yourself. Try not to consume anything with sugar.” She just told me things like this, she did not make me feel I should be scared. I started drinking my coffee without sugar, no soda, just water. I did it and now I think my sugar [levels are] fine.
Participant 170, HbA1c % 5.5, 0% of network diabetic	Well, sometimes my dad will tell me that I should lose weight because right now he is predisposed to diabetes. So, he will tell me “You have to take it easy with your *comidita* (food).”
Participant 127, HbA1c 5.4%, 0% of network diabetic	[This alter, a woman from the neighborhood] does not consume any sugar, walks a lot and she is not fat. She is a very slim woman [despite being diabetic]. Well, this woman does not eat out only in her house. You never see her at a restaurant. She does not eat out because she says she prefers her own [homemade] tortillas with salsa. She says that when you eat out you do not know what they put in the food, so she prefers to eat her own tortillas with salsa and does not spend the money.

### Role of medical doctors and other factors in diabetes management

Although participants acknowledged the importance of social networks members for support with anything from housing issues to making doctors’ appointments, they also emphasized the authoritative role of medical doctors in regards to their health and diabetes management specifically. Some of the illustrative quotes specifically used the words like “authority” and suggested that only medical doctors had the power to make decisions about their health even if they had a supportive network. In some cases, participants questioned the validity of the health information provided by network members, and in others they struggled with other diabetic alters on best approaches to diabetes management. For example, participant (ID 17) emphasized the importance of stress for her and how it affected her mood affected adherence to diabetes medication, but found it hard to convince others in her network of this connection. On the other hand, participant (ID 134) had a supportive alter, her husband, who actually accompanied her to her doctor’s appointment and helped alleviate her guilt about taking time off work to do this. Irrespective of level of support from the network, however, none of the participants actually spoke about using Hba1c levels as a metric to assess their health and well-being in their lives, or the extent to which they spoke to their doctor about this.

**Table:** 

Participant 17, HbA1c 6.3%, 15% of network diabetic	I have noticed this in other people with diabetes and they tell me sometimes they take their pills and sometimes they do not. I have normal [HbA1c] levels and I would ask myself “do I feel tired?” “Is it that I feel obligated to take this medication?” I did not know it then, but it was stress. But [these same diabetics] tell me “I feel this and I feel that, but sometimes I just want to cry.” I tell them this is stress, but they tell me “No this is not stress, it is just the disease that I have [diabetes].” And it is true there are times that you really do not have the will to take your medication…to do anything, [including] eating. But I think this is why stress also matters because I am always running around…picking up my [child] and taking him from here and there. I go to the market but I [am also running around] and there is no time to eat and prepare [food] so you end up picking easy things like ham and other cold cuts…it is just what is easiest.)
Participant 104, HbA1c 5.9%, 5% of network diabetic	For [diabetes] information, I think a doctor is better. I always try to go to the doctor for that type of thing. I will go [to some alters] for things like when I had an issue with apartment. But if it [is] related to an illness, I think they would give me bad information starting with "here, take this, go there." When I say something like "something hurts me here" right away they give me a pill. I have to tell [them] no.
Participant 88, HbA1c 6.7%, 15%	Only my doctor can tell me what to do. Only [my doctor] has the authority to nag me [about my health].
Participant 134, HbA1c missing, 10% of network diabetic	Yes, these [alters] may help orient me. But I always go to my doctor for [things related] to my diabetes.
Participant 134, HbA1c missing, 10% of network diabetic	[My husband] would tell me “Do not miss your appointment…forget work. You always have to make it to your doctor. Miss [work]; you have to go. Just forget work, you know you are diabetic, so c’mon. And he would go with me. He would say, let’s both go to the doctor…just forget work and go to the doctor.

## Divergent group: Normal HbA1c levels with higher percentage of alters and high HbA1c levels and a low proportion of people in their network with diabetes

The participants who lacked a single member with diabetes in their network but had an elevated Hba1c level of 6.3% described close relationships with friends with whom they would often have meals or snacks. While their friends encouraged healthy eating and provided general health support, the participant’s family had a greater influence on what they ate at home. When asked about support for healthy eating, they described their mother “as always cooking for the family” and their father as the person who ultimately paid for all the food purchases in the home. The father’s recent health diagnosis had caused significant changes in how food was purchased and cooked in the household. The participant did not seem to be negotiating much about their diet at home, but they said, "Because my mom is always kind of looking out for what we eat at home, then like, when I am outside I kind of treat myself.” In other words, their autonomy over food was largely preserved outside the home. However, they did not evaluate the foods they consumed outside of the home as relevant to their own health, including their elevated Hba1c levels. Despite this, this participant had a largely positive evaluation of their overall health.

The other divergent participant with an HbA1c level of 4.7% had a support network mostly comprised of friends who were members of a Herbalife health club, a supplement company that has gained popularity among Latinos in the U.S and Latin America. The participants evaluated and interpreted their behavior through the prism of this cluster in their network and described how it helped them adopt healthier eating practices and increase their physical activity. Although the participant acknowledged that they could do more to improve their health, any health concerns were largely focused on weight rather than their Hba1c levels.

## Discussion

Understanding the impact of social relationships on the etiology and management of diabetes is particularly important for immigrant Mexicans, who have the highest prevalence of the disease among all Latino subgroups [1, 3, 78]. Although past research has identified various individual-level factors that correlate with diabetes prevalence among Mexicans and Latinos, such as the locus of control orientation and acculturation [18, 27], few studies have focused on network-level factors [17]. Therefore, investigating how social relationships shape diabetes-related behaviors and outcomes among this population has significant public health implications.

Our study found an association between increased HbA1c levels and diabetic network members. Qualitative data highlighted that participants framed their glycemic control based on whether they were in an acute state of “sufrimiento” or pain. Those who experienced physical discomfort but, due to not being in such an acute state, could still fulfill their work or caretaking duties in their everyday lives, felt that glycemic control did not warrant further attention, including seeking medical care. To the extent that these values are also widely held by the personal network that people are embedded in (i.e., those alters who are diabetic), warrants further investigation if personal networks are to be harnessed as part of the intervention strategy. Other studies among Mexican Americans with T2DM have also found that energy levels and mood are salient symptoms in used to evaluate T2DM management [79]. Our findings emphasize the need for diabetes education that focuses on glucose monitoring is crucial, as it has been found to improve short-term T2DM outcomes among Mexican Americans [6], and may incorporates personal networks (e.g., diabetic alters in the network).

Other network-level factors were associated with HbA1c though not at the same level of statistical significance. Given the pilot nature of this study, we think it is still worth reflecting on alters who encouraged ego health were the most significant network-level variable associated with decreased HbA1c levels. Specifically, an increase in the proportion of alters who encouraged the ego’s health was associated with a decrease in Hba1c levels. The qualitative findings further shed light on the fact that network members who encouraged the ego’s health mostly did so by sharing knowledge and advice regarding the diet. Participants mentioned that reducing sugar and red meat intake, reducing food portions, and eating out less were the most salient diet-related information that flowed through the networks. Our findings are consistent with previous qualitative research among Mexican Americans, which suggests that a diet high in sweets is understood as a “provoking behavior” that causes diabetes (18). Sugar is highly predictive of a distinct dietary pattern among Mexican Americans across the acculturation continuum (75) and “heritage-specific” dietary patterns (76). However, more research is needed to fully understand the diffusion dynamics of dietary information among Mexican Americans. This includes investigating whether efforts by social network members influence social control (e.g., monitoring) or a combination of control and social support, as others have found (77).

Furthermore, future research should investigate the extent to which the diffusion of dietary information across networks among Mexican Americans is associated with specific T2DM outcomes and symptoms. It is important to understand how those in the “middle” phase of the acculturation process think about and practice healthy eating, and how these may adhere to segmented patterns of assimilation rather than purely linear approaches (24). Additional research is necessary to understand these processes at the network level since the qualitative and quantitative findings suggests this may matter for glycemic control.

Finally, our study found individual-level variables associated with decreased levels of Hba1c, namely body mass index and the health eating index total score. These findings are consistent with previous research among both Latinos and non-Latinos that show diets that score highly on the HEI are associated with a significant reduction in the risk of T2DM [80, 81]. Indeed, the qualitative narratives focused on participant’s concepts of a healthy diet, though we did not directly address how these concepts deviated from the way in which HEI is derived during the interviews. Our quantitative results regarding BMI highlight the complex relationship between this anthropometric measure and HbA1c [82], though the qualitative narratives did make clear that reaching an ideal weight was difficult for most participants irrespective of their HbA1c score. Interestingly, a major qualitative theme was the extent to which medical doctors were regarded as the ultimate authority on T2DM information and advice, and not other alters in their network (e.g., family, friends). Future social network interventions should explore the possibility of including physicians as part of the web of social resources for Mexican Americans with T2DM, or at the very least incorporate effective ways in which participants could harness the clinical encounter for the purposes of increasing their diabetes awareness and control [78].

While our study provides valuable insights into the social and individual determinants of Hba1c levels among Mexican Americans with T2DM, several limitations should be considered. First, our study is its observational nature precludes us from making causal claims at the individual or network level. However, our findings are consistent with previous research, lending credibility to their potential causal associations. Second, our sampling strategy was non-probabilistic, in the sense that the sample was derived from a specific community in New York City and may not represent important segments of the entire Mexican American population living in the United States. Future research should replicate our study in other Mexican American communities to assess the extent to which our results generalize to other populations. In a related manner, our data were collected before the 2019 COVID-19 pandemic, which may further limit the generalizability of our results, depending in part on how the pandemic progresses in the future. Therefore, caution should be exercised in generalizing our findings to the current context. Third, our reliance on a single measurement of Hba1c is atypical, and in clinical practice, would require confirmatory testing. These can include either the same or a different test on a different day or a different test using the same blood sample [83]. Fourth, a criticism of personal network research is that it is limited by virtue of obtaining only the view from the ego [43], so discrepancies in perceptions between the ego and the alter are missed. However, perceptions are still critical components in understanding social connections (e.g., perceptions of social support) [84] and these, along with structural characteristics, are key elements in our conceptual framework. Fifth, the mixed method nature of our study limited our capacity to employ more in-depth qualitative methods, such as ethnographic work that necessitates years in the field. Further, integrating social network visualizations into the in-depth qualitative interviews entailed asking highly specific questions about each alter, a departure from the typical in-depth interview format within the interpretative phenomenology framework. Yet it is important to note that methodological applications of this and other qualitative approaches can sometimes suffer from disciplinary silos [85], and perhaps those outside public health might interpret these deviations in our qualitative approach as a flaw. Future research should address the tensions between the qualitative and quantitative arms of the study as they emerge, while also considering the extra resources needed to integrate different methods effectively. It would also entail cross-collaborations between scholars in different fields that others have called for [85] in order to foster an appreciation for innovative, mixed-method work.

Despite these limitations, our mixed-method study provides critical evidence on the role of social networks in T2DM outcomes in an important Latino subgroup, which is often aggregated in national surveillance efforts. Mexican Americans represent the largest and fastest growing subgroup in the United States and are disproportionately burdened by T2DM. Researchers argue that this disease may erode the health advantages seen in this group among immigrant generations in other health areas [86]. Our study addresses a significant gap in the research on Mexican Americans by focusing on network processes that may be crucial to understanding the well-documented disparities in T2DM-related outcomes experienced by this population.

## Supporting information

S1 Checklist COREQ Checklist. (PDF)

S1 Table Summary of regression model diagnostics. (DOCX)
