# Supplementary material for: “No sufro, estoy bien/I am not suffering, so I am doing OK”: A mixed method exploration of individual and network-level factors and Type 2 Diabetes Mellitus (T2DM) among Mexican American adults in New York City
Source: PLoS One. 2024 Jan 19;19(1):e0295499. doi: 10.1371/journal.pone.0295499 (PMC10798639; doi:10.1371/journal.pone.0295499)
Supplement: S1 Table — (DOCX) [file pone.0295499.s002.docx]

**Table S1**: Summary of regression model diagnostics

| **Model assumption** | **Diagnostic results** | | **Conclusion** |
| --- | --- | --- | --- |
| Homoscedasticity of residuals | 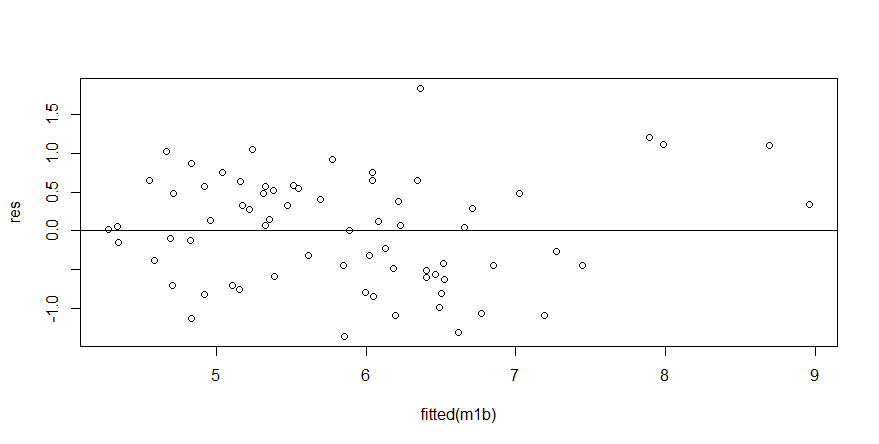Plot of residuals versus fitted values | | The residuals appear homoscedastic. |
| Normal distribution of residuals | 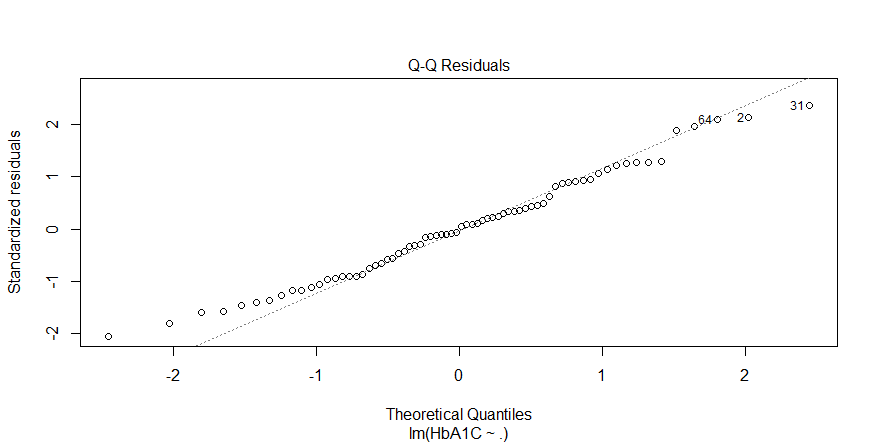Quantile-quantile plot of standardized residuals versus theoretical quantiles | The residuals appear normally distributed. | |
| Outlier detection | Bonferroni p-values | No studentized residuals with Bonferroni p-value less than 0.05. | |
| Multicollinearity | Variance inflation factor (VIF) | No multicollinearity as all of the VIFs were less than the tolerance of 5.0. | |
